# Trends of sensitization to aeroallergens in patients with allergic rhinitis and asthma in the city of Bursa, South Marmara Sea Region of Turkey

**DOI:** 10.3906/sag-1908-139

**Published:** 2020-04-09

**Authors:** Dane EDİGER, Fatma Esra GÜNAYDIN, Müge ERBAY, Ümmühan ŞEKER

**Affiliations:** 1 Section of Immunology and Allergy Diseases, Department of Chest Diseases, Faculty of Medicine, Uludağ University, Bursa Turkey; 2 Section of Immunology and Allergy Diseases, Department of Dermatology, Bursa City Hospital, Bursa Turkey

**Keywords:** Aeroallergen, asthma, allergic rhinitis, Bursa, Turkey

## Abstract

**Background/aim:**

Allergic rhinitis (AR) and asthma are the most common allergic disorders worldwide. Aeroallergens are critical causative factors in the pathogenesis of these disorders and sensitization to aeroallergens differs in various countries and regions. Identification of the most common aeroallergen sensitization is crucial in the diagnosis and management of AR and asthma. We examined the distribution of aeroallergen sensitizations detected by skin prick tests (SPTs) in adult patients with AR and/or asthma in the city of Bursa.

**Materials and methods:**

Five hundred forty-five patients who underwent a SPT and were diagnosed with rhinitis and/or asthma in the Uludağ University Faculty of Medicine’s Department of Immunology and Allergic Diseases Outpatient Clinic from March 2018 to August 2018 were retrospectively evaluated. SPTs with standard extracts including house dust mites, pollens, molds, animal dander, and latex were performed for patients.

**Results:**

A total of 545 patients were included and most of the patients (270; 49.5%) were between 30 and 49 years of age. The prevalence of atopy was 57.9%. The most common aeroallergens detected in SPTs were *Dermatophagoides farinae* (50%) and *D. pteronyssinus* (44%), followed by grass-rye mix (43%), grass mix (38.6%), olive (33.2%), and wheat (32.3%). The sensitization to olive pollen was higher in cases of mild asthma (52%), while sensitization to *D. farinae* was higher in patients with mild and moderate asthma (54.5%, 54.2%) (P < 0.05).

**Conclusions:**

Our study revealed that house dust mite was the most common sensitizing aeroallergen in patients with AR and asthma while pollens were the most common allergen in patients with only AR. The sensitization to grass and olive pollen was higher in cases of mild asthma than moderate and severe. Regional allergy panels may provide important clinical clues for characteristics and courses of allergic diseases.

## 1. Introduction

The prevalence of allergic rhinitis (AR) and asthma has risen significantly over the last two decades [1]. Prevalence of respiratory allergies (AR, asthma) was documented as 12%–20% worldwide [2]. AR is the most common atopic disease and affects more than 500 million people around the world [3]. Furthermore, 300 million people worldwide suffer from asthma, and it is projected to increase to 400 million by the year 2025 [4]. In particular, increasing industrialization and air pollution are considered as leading causes of the high prevalence of AR and asthma [5,6].

The etiologies of AR and asthma are complex, resulting from genetics [7,8] and interacting environmental factors [9,10]. Allergic sensitization to aeroallergens is a major risk factor for developing an allergic disease and optimal management of allergic diseases requires the identification of the allergic sensitivities of the patient [11]. The common allergens include house dust mites (HDMs), grasses, trees, weed pollens, animal dander, and molds [5].

Turkey is a bridge between Europe and Asia, surrounded by the Marmara Sea, Black Sea, and Mediterranean Sea on three sides. In Turkey, the prevalence of AR and asthma varies between 11.4% and 22.7% and between 2% and 17%, respectively, in adult patients based on regional prevalence [4,12–17]. Depending on the considerable variety of climates, aeroallergen spectra and disease prevalence rates differ in distinct regions of Turkey. There is only one study that revealed the aeroallergen sensitivity of patients with asthma in Bursa. Bursa is located to the southeast of the Marmara Sea in Northwest Turkey [18] at an altitude of 100 m above sea level, with average relative humidity of 69%. Because the humidity rate of Bursa Province is above 50%, it facilitates the growth of mites in indoor environments. 

Avoiding exposure to an allergen is the best way to prevent the disease. Our study aims to find the prevalence of various allergens leading to AR and/or asthma through skin prick tests (SPTs) in the city of Bursa, Turkey.

## 2. Materials and methods

This retrospective clinical study was conducted in the Department of Immunology and Allergic Diseases, Uludağ University Medical Faculty Hospital, Bursa, Turkey. The study was approved by the institutional ethics committee of Uludağ University (identification 2019/20).

### 2.1. Subjects

The medical records of 545 adult patients who were diagnosed with rhinitis and/or asthma and lived in Bursa were analyzed. AR and asthma were diagnosed and classified based on the Allergic Rhinitis and Their Impacts on Asthma (ARIA) and the Global Initiative for Asthma (GINA) guidelines, respectively. Asthma severity was classified as mild/moderate/severe according to medication use with level of treatment as defined in GINA as mild (steps 1–2), moderate (step 3), and severe (steps 4–5) [19]. We reviewed the medical records of all patients who presented to our department from March 2018 to August 2018.

### 2.2. Skin prick tests

The SPT was conducted using a standard commercial extract panel (Alk-Abello, Lincoln Diagnostics, Dallas, TX, USA), consisting of 17 aeroallergens (grass mix, grass-rye mix, weed mix, birch, trees mix, olive, oak, wheat, cat, dog, latex, *Dermatophagoides pteronyssinus*, *D. farinae*, *Acarus siro*, *Fusarium*, *Alternaria*, cockroach). SPT was performed according to the international guidelines as a single test on two forearms with lancets and standardized allergens by the same trained nurse. Histamine hydrochloride (10 mg/mL) and 0.9% saline were applied as positive and negative controls, respectively. The wheel diameter was measured after 20 min and reported in mm. A skin reaction of at least 3 mm greater than that produced by the negative control in the SPT was considered as a positive reaction.

### 2.3. Analysis

The data were analyzed using SPSS 21 (IBM Corp., Armonk, NY, USA). Chi-square tests, independent t-tests, and Mann–Whitney U tests were used to examine and compare the relationship between the characteristics of the sample. P < 0.05 was considered statistically significant.

## 3. Results

### 3.1. Study population

A total of 545 patients (407 female and 138 male) were enrolled in this study. The median age was 41 years (range: 18–82). Most of the patients (270; 49.5%) were in the age group of 30–49 years (Table). Three hundred sixteen of the patients (57.9%) were found to be sensitized to at least one of the 17 aeroallergen extracts tested. One hundred sixty-seven of the sensitized patients (52.8%) had AR without asthma, 135 of the sensitized patients (42.7%) had AR with asthma, and 14 of the sensitized patients (4%) had only asthma.

### 3.2. Prevalence of aeroallergen sensitization

**Figure 1:**
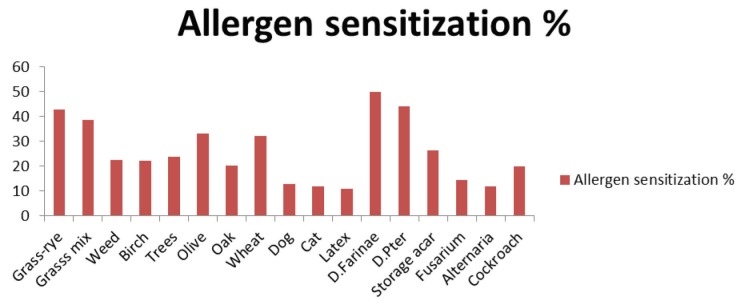
Distribution of allergen sensitization of the study population.

The most frequent aeroallergens that we determined in the SPTs were mites (*D. farinae* 50%; *D. pteronyssinus* 44%), followed by grass-rye mix (43%), grass mix (38.6%), olive (33.2%), wheat pollen (32.3%), storage mite (*Acarus siro* 26.3%), tree pollens (23.7%), weed pollen (22.5%), birch pollen (22.2%), oak (20.3%), cockroach (19.9%), *Fusarium *(14.6%), dog (12.7%), cat (12%), *Alternaria* (12%), and latex (10.8%) (Figure 1).

### 3.3. Sex and allergen sensitization

Females showed higher test positivity rates for grass-rye mixes, weeds, olive pollen, and cats compared to males (P = 0.047, P = 0.017, P = 0.034, and P = 0.049, respectively). No significant difference was observed between the sexes for the remaining allergens. 

### 3.4. Age groups and allergen sensitization to pollens

We classified patients into three groups according to age cutoff points commonly used in the medical literature: 18–29, 30–49, and 50+ years old. There was a significant difference among these groups for grass-rye mix, grass mix, and wheat allergen sensitivity rates. The sensitization rates to the grass-rye mix were 32.4%, 55.1%, and 12.5% for subjects of 18–29 years, 30–49 years, and 50+ years, respectively. We found a significant association between sensitization to grass-rye mix and the age groups of the subjects (P = 0.001). The sensitization rates against grass were higher in the group of 30–49 years (53.3%) than 18–29 years (33.6%) and 50+ years (13.1%) (P = 0.001). The prevalence of sensitization to wheat was also higher in the group of 30–49 years (55.9%) than 18–29 years (33.3%) and 50+ (10.8%) (P = 0.001).

**Table 1:** Distribution of all the patients and the patients with positive and negative SPTs according to age, sex, and diagnosis.

	Positive SPT(n = 316)	Negative SPT(n = 229)	P-value
Age (median ± IQR)	38.5 ± 21	43 ± 20.5	0.059
Age groups			
18–29 years (n)	81	33	0.000
30–49 years (n)	154	116	0.021
50+ years (n)	81	80	0.937
Sex (female/male)			0.010
	22393	18445	0.0530.000
Asthma (female/male)FemaleMale	11137	10725	0.7860.128
Rhinitis (female/ male)FemaleMale	21092	15540	0.0040.000

SPT: Skin prick test.

### 3.5. Asthma and/or allergic rhinitis and common allergen sensitization

In asthma patients with AR, the most frequent aeroallergens were mites (*D. pteronyssinus* 52.6%; *D. farinae* 51.3%), followed by grass-rye mix (41.5%), grass mix (38.5%), olive pollen (31.1%), storage mites (28.1%), wheat pollen (28.1%), cat (11.2%), and dog (14.1%).

In patients with only AR, the most frequent aeroallergen was grass-rye mix (52.8%), followed by mites (*D. farinae* 44.3%; *D. pteronyssinus* 40.1%), grass mix (39.5%), olive pollen (36.5%), wheat pollen (36.5%), weed pollen (25.1%), and storage mites (25.1%). Latex (8.4%) and *Alternaria* (9%) were rare.

**Figure 2:**
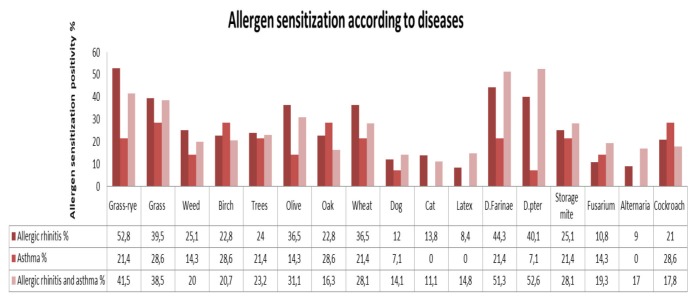
Distribution of allergen sensitization according to diseases.

In patients with only asthma, the most frequent aeroallergens were grass mix (28.6%), birch (28.6%), oak (28.6%), and cockroach (28.6%), followed by grass-rye mix (21.4%), storage mites (21.4%), *D. farinae* (21.4%), wheat pollen (21.4%), and tree pollen (21.4%). Cat, latex, and *Alternaria* sensitizations were not seen (0%) (Figure 2).

### 3.6. Asthma severity and common allergen sensitization

**Figure 3:**
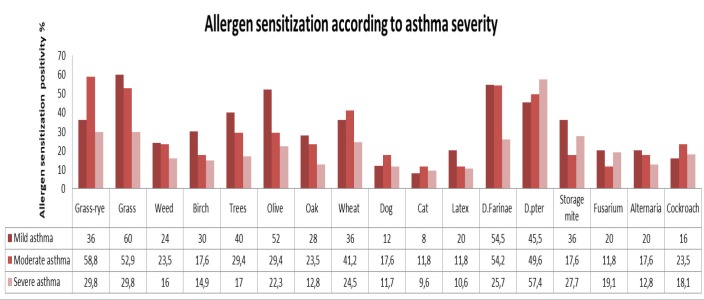
Distribution of allergen sensitization according to asthma severity

Our study showed an association between sensitization to four aeroallergens (grass-rye mix, grass mix, olive pollen, *D. farinae*) and asthma severity. The sensitization rates to the grass-rye mix were 36%, 58.8%, and 29.8% for subjects with mild, moderate, and severe asthma, respectively. The sensitization rates against grass were higher in cases of mild asthma (60%) than moderate (52.9%) and severe (29.8%) (P = 0.01). The sensitization to olive pollen was higher in cases of mild asthma (52%), while sensitization to *D. farinae* was higher in patients with mild and moderate asthma (54.5%, 54.2%) (P = 0.04) (Figure 3).

## 4. Discussion

AR and asthma are leading causes of morbidity around the world, and the prevalence of these diseases is continuously increasing related to growing environmental pollution [3,6]. Identification of allergens that trigger factors in AR and asthma is the initial step, and the treating physician should be aware of the geographical distribution and prevalence of aeroallergens in a particular area [20]. The SPT is an easy and quick tool for IgE-mediated allergy diagnosis. As far as we know, this is the first reported study to investigate the aeroallergen distribution in adult subjects with AR and/or asthma to the southeast of the Marmara Sea, in Northwest Turkey.

Turkey, a country having lands both in Asia and Europe, consists of many different geographic features and climates. Turkey is surrounded by sea in three directions, having a rainy climate in the north, a Mediterranean climate in western and southern regions, and a cold and rough climate in the eastern and inner parts. The western and northwestern parts are industrialized [15]. These geographical and climatic differences result in varying aeroallergen spectra and subsequently different sensitization rates to these aeroallergens [6]. Bursa is an industrialized city with three million inhabitants, situated at 41°11′N, 29°04′E in northwestern Turkey at an altitude of about 100 m above sea level, located on a plain flanked by Mount Uludağ to the south and the Samanlı Range to the north [21].

In our study, 316 patients had a positive reaction to the SPT. HDMs were the most prevalent sensitizing aeroallergens (*D. pteronyssinus* 50%; *D. farinae* 44%), followed by pollen (grass-rye mix 43%, grass mix 38.6%, olive 33.2%, wheat 32.3%, trees mix 23.7%, weeds 22.5%, birch 22.2%, oak 20.3%), storage mites (26.3%), cockroach (19.9%), molds (*Fusarium* 14.6%, *Alternaria* 12%), and epithelia (dog 12.7%, cat 12%). There is remarkable variability in aeroallergen distribution among countries, and even among regions within the same country [4]. In general, HDM sensitization is more common in tropical countries such as Malaysia and Singapore [22]. However, pollens are the most sensitizing aeroallergens in Europe [23]. Allergic sensitization to mites has been studied in different regions of Turkey. Studies in Turkey have reported sensitization rates to *D. pteronyssinus *and *D. farinae* as 72.5% and 63.7% in Düzce [24], 62.2% and 51.3% in Eskişehir [25], 25.3% and 29.3% in İstanbul [26], and 22.4% and 21.5% in Isparta [27]. A previous study that included various regions of Turkey showed that the presence of mites was related to an increase in both mean temperature (>15 °C) and humidity (³40%), as well as low altitude (<300 m) [28]. In our study, high sensitization to HDMs was expected due to the regional geography with 69% humidity and a location at an altitude of 100 m above sea level.

Our study showed more prevalent sensitivity to grass-rye mixes, weeds, olive pollen, and cats among women. Conversely, other studies from Kuwait and China showed a higher prevalence rate of sensitization towards outdoor allergens in males [29,30]. No convincing clarifications have been given for these differences between the sexes. There is no evidence to suggest that sex leads to differential exposure to aeroallergens [29].

Looking at the age distribution of the allergen groups, sensitization to grass-rye, grass, and wheat were seen more frequently in patients between 30 and 49 years. We found no difference in sensitization to other aeroallergens according to age groups. A previous study from Kuwait indicated age-dependent sensitization to the HDM *D. microceras*, which had a higher prevalence rate in the 45+ years age group [29]. Other studies from Turkey and other countries found no difference in the prevalence of positive SPT between younger and older patients with AR [26,27,31].

HDM sensitization associated with the risk of rhinitis and asthma in children and adults has been shown by several researchers [32,33]. Allergic responses to HDM are associated with airway hyperresponsiveness and it is the most important indoor allergen for asthma [34–37]. A previous study from Bursa and other studies showed high sensitization to HDM in asthma patients [5,17,32,38]. In accordance with the literature, we found high HDM sensitivity in asthmatic patients. Related to the regional geography with high humidity and temperature, sensitization to indoor allergens was expected.

Looking at the association between asthma severity and allergen sensitivity, olive and grass sensitization was higher in cases of mild asthma. It is known that 24.4% of olive trees in Turkey are located in the Marmara region, and 37.4% of the olive trees in the Marmara region are in Bursa. In addition, the most commonly grown fruit in the province is olive [39]. Therefore, sensitization to olive tree is expected. *D. farinae* sensitization was higher in cases of mild and moderate asthma. Grass-rye sensitization was higher in cases of moderate asthma. A study from Kuwait indicated a high frequency of severe asthma in patients sensitized to *Cladosporium*, *Aspergillus*, and *Alternaria*. A study from Saudi Arabia showed higher asthma severity correlation with the degree of sensitization to *D. pteronyssinus* and *D. farinae*.

For patients with only AR, the most frequent aeroallergen was the grass-rye mix, followed by mites, grass mix, and olive. In this regard, our results are consistent with some previous studies, which indicated grass pollens as the most common allergen in patients with AR [26,29,40,41]. On the other hand, some previous studies from many countries showed that HDMs were common aeroallergens in patients with AR [4,5,31,40–42]. This appears to be a geographical variation. Our findings correlated with the climate of Bursa, which is characterized by dry and warm summers and wet and cold winters. The climate in Bursa allows many plants to grow, flower, and produce and release pollen grains into the atmosphere at any time of the year. The number of pollen grains increases from February to April and reaches its maximum level in May [21]. Grass pollen, which is the most dominant aeroallergen in May and June, causes great problems for individuals with pollen allergies [21,43].

In conclusion, the most frequent aeroallergen that we determined in the SPT was mites, followed by pollens, cockroach, mold, animal dander, and latex. Our study showed high HDM sensitization in patients with asthma and AR; on the other hand, sensitization to pollens was most common in patients with only AR. Furthermore, olive-sensitive patients had mild asthma, and mite-sensitive patients had mild to moderate severity. Identification of allergens is an essential step in the diagnosis of respiratory allergic disease. Specific allergy panels based on regional differences in patterns of sensitization can provide cost-effective screening of sensitized patients and better management of the disease with environmental allergen control.

## Acknowledgments

The authors would like to thank allergy clinic nurses Meral İnal and Mürüvvet Canık for performing and archiving the skin prick tests.
